# Epidemiological Characteristics and the Dynamic Transmission Model of Dengue Fever in Zhanjiang City, Guangdong Province in 2018

**DOI:** 10.3390/tropicalmed7090209

**Published:** 2022-08-25

**Authors:** Meng Zhang, Jie-Feng Huang, Min Kang, Xing-Chun Liu, Hong-Yan Lin, Ze-Yu Zhao, Guo-Qiang Ye, Sheng-Nan Lin, Jia Rui, Jing-Wen Xu, Yuan-Zhao Zhu, Yao Wang, Meng Yang, Shi-Xing Tang, Qu Cheng, Tian-Mu Chen

**Affiliations:** 1 Guangdong Provincial Center for Disease Control and Prevention, Guangzhou 511430, China; 2 State Key Laboratory of Molecular Vaccinology and Molecular Diagnostics, School of Public Health, Xiamen University, Xiamen 361102, China; 3 School of Public Health, Southern Medical University, Guangzhou 510515, China; 4 Zhanjiang Municipal Center for Disease Control and Prevention, Zhanjiang 524037, China; 5 Division of Environmental Health Sciences, School of Public Health, University of California, Berkeley, CA 94704, USA

**Keywords:** dengue fever, epidemiology, mathematical model, transmissibility

## Abstract

**Background:** With the progress of urbanization, the mobility of people has gradually increased, which has led to the further spread of dengue fever. This study evaluated the transmissibility of dengue fever within districts and between different districts in Zhanjiang City to provide corresponding advice for cross-regional prevention and control. **Methods:** A mathematical model of transmission dynamics was developed to explore the transmissibility of the disease and to compare that between different regions. **Results:** A total of 467 DF cases (6.38 per 100,000 people) were reported in Zhanjiang City in 2018. In the model, without any intervention, the number of simulated cases in this epidemic reached about 950. The dengue fever transmissions between districts varied within and between regions. When the spread of dengue fever from Chikan Districts to other districts was cut off, the number of cases in other districts dropped significantly or even to zero. When the density of mosquitoes in Xiashan District was controlled, the dengue fever epidemic in Xiashan District was found to be significantly alleviated. **Conclusions:** When there is a dengue outbreak, timely measures can effectively control it from developing into an epidemic. Different prevention and control measures in different districts could efficiently reduce the risk of disease transmission.

## 1. Introduction

Dengue fever is an important mosquito-borne (mainly by *Aedes* mosquitoes [1,2,3]) viral disease that is caused by four different serotypes of the dengue virus (DENV 1-4). According to the World Health Organization (WHO), the incidence rate of DF around the world has grown sharply and dengue fever was also listed as one of the potentially threatening diseases in 2019 by the WHO [4]. Dengue could be widespread in tropical and subtropical regions around the world when given favorable temperature, precipitation and humidity conditions [5]. Studies showed that temperature greatly influences *Aedes albopictus* mosquitoes, which are the vector of dengue fever. Having a minimum temperature above 18 °C is one of the conditions suitable for the *Aedes* mosquitoes to breed and thus develop a DF epidemic [6].

Guangdong province is located in the south of China and has a subtropical climate, while Zhanjiang City is one of the prefecture-level cities of Guangdong Province. Since the first outbreak in Guangdong Province in 1978, dengue cases have been reported for years [7], and Guangdong has become the region with the highest incidence rate of DF in China since then [8]. *Aedes albopictus* mosquitoes, which are densely distributed throughout Guangdong province, are considered the main vector [9]. The dengue serotypes detected in Guangzhou were mainly DENV 1 and DENV 2 [10]. Between 2008 and 2018, more than 85% of indigenous DF cases in China occurred in Guangdong Province [11]. In June 2014, an unprecedented DF outbreak occurred in Guangdong Province. A total of 14,594 clinically diagnosed and 11,387 laboratory-confirmed cases were reported, more than any of the DF epidemics in China in the past 20 years [12,13].

Many mathematical models are available for studying dengue fever. Lan Zhou [14] designed a dengue model with vertical transmission and the results showed that if we could control mosquito population growth, dengue could be eliminated. A dengue transmission model that considered time-dependent entomological parameters and assessed seasonal variations in temperature and precipitation during dengue epidemics was created [15]. However, this research focused on the media and ignored the human impact. There was an SIR model that was created to describe the vector-to-host and host-to-vector transmission dynamics of dengue, as well as the optimal time for pesticide use [16]. However, the impact of host mobility on transmission was not considered. G Bhuju et al. used an SEIR-SEI model to explore the effect of dengue viral load on the transmission dynamics of dengue disease through fuzzy behavior [17]. One study explored the role of asymptomatic infection in the epidemiology of the dengue virus through the susceptibility, asymptomatic, infection and recovery (SAIR) model [18]. In Thailand, a national study that revealed the spatial and seasonal patterns of dengue fever at the regional level was conducted and tested whether the peak of dengue fever in Bangkok was ahead of the peak of dengue fever in other provinces in Thailand [19]. However, it has not yet been considered whether there is a mutual transmission of dengue fever in space. This study used the data on dengue fever in Zhanjiang City in 2018 to investigate the mutual transmission of dengue fever between the host and the vector. At the same time, the mobility of hosts between different districts, which causes disease transmission between regions, was taken into consideration.

In order to provide corresponding suggestions for cross-regional prevention and control in Zhanjiang City and to offer methods for studying the trans-regional transmission of DF in other regions, we developed an ordinary differential equation (ODE) model to simulate the transmission dynamics of the dengue virus in this study. This was the first study that used the SEI-SEIAR model to explore the dynamics of dengue fever spreading between districts.

## 2. Methods

### 2.1. Materials

In 2018, the first dengue outbreak occurred in Chikan District, followed by outbreaks in Xiashan District, Mazhang District and Kaifa District, all of which are geographically adjacent to Chikan District. Because of the onset time lag of patients between Chikan and the other three districts, we suspected that the disease was transmitted from one region to another. Therefore, we examined the transmissibility between Chikan District and the other three regions. The second indigenous case in Chikan District occurred on 27 August. Since then, the number of cases has gradually increased and peaked on 27 September. At this time, Zhanjiang CDC implemented intervention measures, but the outbreak in Chikan District continued until 6 October. We identified the index case as the first indigenous case in Chikan District. Since there were only two indigenous cases in Potou District, we omitted this district in our analyses. The last indigenous case in the study districts was reported on 28 October in Xiashan District.

We collected data on the daily cases from 17 August to 28 October for the simulation. In addition, we developed a susceptible–exposed–infectious/asymptomatic–removed (SEIAR) model and a susceptible–exposed–infectious (SEI) model for humans and mosquitoes, respectively, to explore the transmissibility of the dengue virus between Chikan district and other districts.

### 2.2. Study Site

In 2018, Zhanjiang consisted of 5 municipal districts (Chikan, Xiashan, Potou, Mazhang and Kaifa Districts), 3 county-level cities (Wuchuan, Leizhou and Lianjiang Cities) and 2 counties (Xuwen and Suixi Counties). The permanent resident population of Zhanjiang City was 7.33 million. The population of Chikan District, which is the center of Zhanjiang City, was about 298,000 people and the land area was 79 square kilometers. Chikan District (110°20′ to 110°21′ E, 21°14′ to 21°19′ N) is located in the tropical northern margin south of the Tropic of Cancer, with an annual mean temperature of 22.8 to 23.5 °C, annual cumulative precipitation of 1596 mm and annual mean sunshine duration time of 1927.7 h. Chikan District is surrounded by Mazhang, Xiashan, Potou and Kaifa Districts. In 2018, the population of Xiashan District was approximately 436,000 people, Mazhang had approximately 275,000 people and Kaifa had approximately 334,000 people. *Aedes* mosquitoes are the only dengue vector species in Guangdong Province.

### 2.3. Data Collection

Data from DF cases and daily vector surveillance were obtained from Zhanjiang Center for Disease Control and Prevention (CDC). General information for each case included gender, age, address and occupation. For a subset of the cases, information about the type of diagnosis (confirmed and clinical), virus serotype, and onset and diagnosis time were also collected.

### 2.4. Case Definitions

DF cases were diagnosed according to the Dengue Diagnosis Criteria (WS 216—2008, WS 216—2018) issued by the Chinese Ministry of Health as follows:

Clinically diagnosed cases: suspected cases of leucopenia or thrombocytopenia, or suspected cases with a positive serum test for IgG or IgM.

Laboratory confirmed cases: suspected cases of dengue virus (DENV) RNA in serum detected using real-time fluorescent quantitative PCR; or the virus was isolated from the blood, tissue or cerebrospinal fluid of an acutely infected person; or the IgG titer in the recovery stage was 4 times higher than that in the acute stage.

Imported cases were defined as dengue cases who had traveled to dengue-endemic districts within 2 weeks before symptom onset, while local cases were defined as those who did not leave Zhanjiang City within 2 weeks before the onset of dengue [20].

According to the Dengue Diagnosis Criteria (WS 216—2008, WS 216—2018) issued by the Chinese Ministry of Health, a DF outbreak is defined as the occurrence of 3 or more indigenous laboratory-diagnosed dengue cases during a maximum incubation period (14 days) in a relatively concentrated population location (such as a community, neighborhood committee, village, school or another collective unit)

### 2.5. Vector Surveillance

During the epidemic season, when imported or local cases of dengue fever infection are found, as an important part of the investigation and treatment of the epidemic, emergency surveillance is activated.

#### 2.5.1. Surveillance Area

Core area: With reference to the activity of *Aedes* mosquito, multiple core areas were divided centered on the infected person’s residence, households adjacent to it, the infected person’s workplace, and other places where the case has been.Alert zone: A radius of 200 m outside the core zone. In rural areas, natural villages and tunnels around the core area, and if necessary, administrative villages or even townships, are set as alert zones. If necessary, administrative villages or even towns or villages should be considered alert zones. In urban areas, the alert area is usually the core area surrounded by some streets, lanes, neighborhood committees or streets.

Surveillance zone: According to the epidemic size and epidemic season of different dengue risk areas, the surveillance zone will be set up outside the vigilance zone. The surveillance area is defined around the alert area.

#### 2.5.2. Monitoring Methods

All dengue mosquito vector emergency monitoring points were monitored using the Breteau index method.

#### 2.5.3. The Frequency of Monitoring

Breteau index method: After 1–2 days of the dengue epidemic, a full coverage survey and emergency mosquito vector control were conducted in the core area, followed by repeated control and survey every 2–3 days until the BI was less than 5. The alert area was surveyed once a week, while the surveillance area was surveyed once every 2 weeks.

#### 2.5.4. Data Analysis and Feedback

Risk assessment: A Breteau index (BI) of less than 5 is the threshold for controlling dengue transmission. A BI greater than 5 has the risk of transmission, greater than 10 has the risk of an outbreak, and greater than 20 has the risk of a regional epidemic, requiring continued cleanup of breeding sites, which should be cleared of adult mosquitoes.

Breteau index (BI) is an index that describes the density of *Aedes* mosquitoes and is calculated as follows:

(1) $$\mathrm{BI}=\frac{\mathrm{Positive}\;\mathrm{Containers}\;}{\mathrm{Properties}\;\mathrm{Searched}\;}\times 100$$

Zhanjiang CDC staff followed the technical guidelines for dengue fever control issued by the Chinese Center for Disease Control and Prevention. Four streets/villages were selected in different geographical locations for each surveillance site; at least 100 residential households were surveyed, small water storage containers in indoor and outdoor plots were examined, and then mosquito larvae in positive containers were collected. To avoid the effect of continuous monitoring on mosquito density, two adjacent monitoring sessions were conducted in different households.

### 2.6. Transmission Model

**Figure 1:**
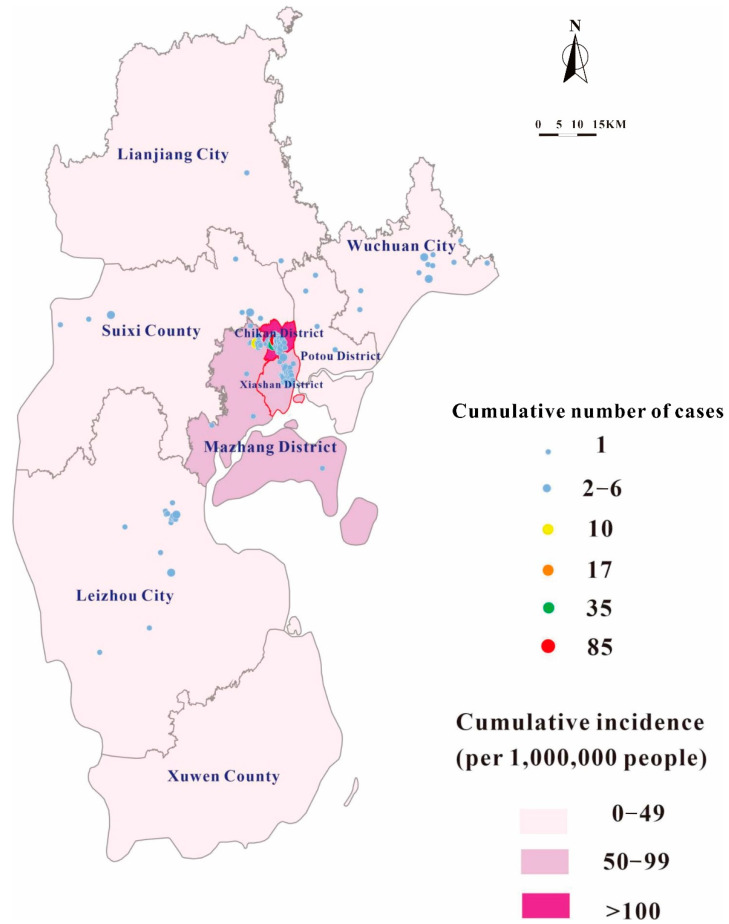
Cumulative number of cases and cumulative incidence of dengue fever in Zhanjiang City in 2018.

The distribution of the DF cases in Zhanjiang City is shown in Figure 1. The darkest district was Chikan District.

In the ODE model based on our previous study [9,21], we found that the outbreak was locally transmitted by an imported case; therefore, we also included the index case in the model. We assumed that an imported case transmitted the virus to the outbreak source, where susceptible mosquitoes in Chikan District became infected with the dengue virus by biting the outbreak source and transmitted the virus by biting susceptible people in Chikan District; then, susceptible individuals may become asymptomatic or symptomatic through an incubation period or latent period. Next, these infected people may be bitten by susceptible mosquitoes in other districts, causing the disease to circulate in different districts. At last, the infected individuals would all recover with immunity. We divided individuals in Chikan District into the following five groups: *S_pc_*—susceptible, *E_pc_*—exposed, *I_pc_*—infectious, *A_pc_*—asymptomatic and *R_pc_*—removed. The subscript *pc* represents people in Chikan District. *I_IS_* represents the imported case that initiated the outbreak. Mosquitoes in Chikan District were divided into three groups: *S_mc_*—susceptible, *E_mc_*—exposed and *I_mc_*—infectious. The subscript *mc* represents mosquitoes in Chikan District. *N_mc_* represents the sum of the mosquitoes in the three mosquito groups. Similarly, individuals in other districts were also divided into *S_po_*, *E_po_*, *I_po_*, *A_po_* and *R_po_*, and mosquitoes were divided into *S_mo_*, *E_mo_* and *I_mo_*. The subscript *po* and *mo* represent people and mosquitoes in other districts, respectively. The definition model parameters are summarized in Table 1.

The mathematical model was described using the following ordinary differential equations:

$$\frac{dI_{Is}}{dt}=-\varepsilon I_{Is}\;\frac{dS_{mc}}{dt}=a_{c}c_{c}\left(N_{mc}-n_{c}I_{mc}\right)-\beta_{Is}S_{mc}I_{Is}-\beta_{pcmc}S_{mc}\left(A_{pc}+I_{pc}\right)-b_{c}S_{mc}\;\frac{dE_{mc}}{dt}=\beta_{Is}S_{mc}I_{Is}+\beta_{pcmc}S_{mc}\left(A_{pc}+I_{pc}\right)-\left(b_{c}+\omega_{m}\right)E_{mc}\;\frac{dI_{mc}}{dt}=a_{c}c_{c}n_{c}I_{mc}+\omega_{m}E_{mc}-b_{c}I_{mc}\;N_{mc}=S_{mc}+E_{mc}+I_{mc}\;\frac{dS_{pc}}{dt}=-\beta_{mcpc}S_{pc}I_{mc}-\beta_{mcpc}S_{pc}I_{mo}\;\frac{dE_{pc}}{dt}=\beta_{mcpc}S_{pc}I_{mc}+\beta_{mcpc}S_{pc}I_{mo}-\omega_{p}E_{pc}\;\frac{dI_{pc}}{dt}=\left(1-q\right)\omega_{p}E_{pc}-\gamma I_{pc}\;\frac{dA_{pc}}{dt}=q\omega_{p}E_{pc}-\gamma^{\prime }A_{pc}\;\frac{dR_{pc}}{dt}=\gamma I_{pc}+\gamma^{\prime }A_{pc}\;\frac{dS_{mo}}{dt}=a_{o}c_{o}\left(N_{mo}-n_{o}I_{mo}\right)-\beta_{pcmo}S_{mo}\left(A_{pc}+I_{pc}\right)-\beta_{pomo}S_{mo}\left(A_{po}+I_{po}\right)-b_{o}S_{mo}\;\frac{dE_{mo}}{dt}=\beta_{pcmo}S_{mo}\left(A_{pc}+I_{pc}\right)+\beta_{pomo}S_{mo}\left(A_{po}+I_{po}\right)-\left(b_{o}+\omega_{m}\right)E_{mo}\;\frac{dI_{mo}}{dt}=a_{o}c_{o}n_{o}I_{mo}+\omega_{m}E_{mo}-b_{o}I_{mo}\;N_{mo}=S_{mo}+E_{mo}+I_{mo}\;\frac{dS_{po}}{dt}=-\beta_{mopo}S_{po}I_{mo}\;\frac{dE_{po}}{dt}=\beta_{mopo}S_{po}I_{mo}-\omega_{p}E_{po}\;\frac{dI_{po}}{dt}=\left(1-q\right)\omega_{p}E_{po}-\gamma I_{po}\;\frac{dA_{po}}{dt}=q\omega_{p}E_{po}-\gamma^{\prime }A_{po}\;\frac{dR_{po}}{dt}=\gamma I_{po}+\gamma^{\prime }A_{po}$$

**Figure 2:**
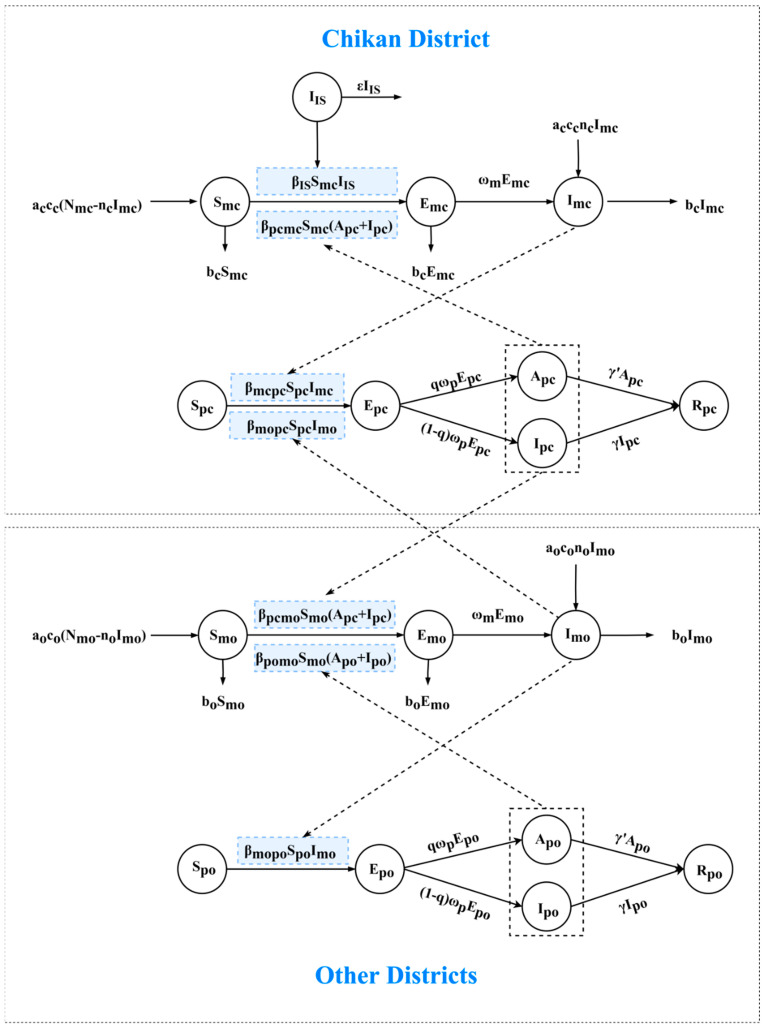
Schematic of the dengue fever transmission model.

Figure 2 shows the schematic of the transmission model.

### 2.7. Parameter Estimation

The values of the model parameters are summarized in Table 1. The season is one of the important conditions that affect the density of *Aedes albopictus* mosquitoes due to the periodicity of DF occurrence; therefore, we introduced the seasonal parameter *c* according to the previous study and used the trigonometric function to simulate the transmission of infection [22]. The incubation period of the dengue virus in humans usually spans from 4 to 8 days; 6 days is considered appropriate, and thus, we used *ω_p_* to represent the incubation relative rate of human infection and defined it as 0.1667 [23]. The extrinsic incubation period (EIP), i.e., the time interval between mosquito infection and when its bites become infectious, is 8–12 days; therefore, we considered 10 days in the simulation, i.e., we used *ω_m_* = 0.1000 [24] to represent the relative incubation rate of mosquito infection. The ratio of symptomatic to asymptomatic infection for DF is generally 2.2:1 [25]; therefore, the proportion of asymptomatic infections was *q* = 0.6875. The infection period is approximately 3–14 days [24]; therefore, the relative removal rate was *γ = γ’ = ε =* 0.1429. Due to the existing similar studies, we referred to the previously published literature to determine that *a_c_ = a_o_ = b_c_ = b_o_ =* 0.0714 [21]. The vertical infection rate of individual positive families (as a percentage of the vertical infection rate of offspring) ranges from 1.4% to 17.4% [26], which was simulated as 10.0% in the model; therefore, *n_o_* = *n_c_* = 0.1000. The calculation of parameters *c_c_* and *c_o_* were as follows, where *τ* and *T* refer to the simulation delay of the initial time in the whole season and the period of the season cycle, respectively:

(2) $$c_{c}=c_{o}=cos\left[\frac{2\pi \left(t-\tau \right)}{T}\right]$$

On 13 September, the Zhanjiang Municipal Government launched a level III emergency response for this public health emergency, set up an emergency command, and established a joint prevention and control mechanism. On 28 September, Zhanjiang City held a dengue fever epidemic analysis meeting to discuss the situation at each epidemic site in Zhanjiang City and the measures taken, study and judge the development trend of the dengue fever epidemic in Zhanjiang City, discuss the implementation of prevention and control measures and the problems at each risk point, and propose a classification for the next step. The government increased its efforts to prevent and control the epidemic. Due to the interventions on 13 September and 28 September, we divided the dengue fever outbreak into three periods (17 August–12 September, 13–27 September and 28 September–28 October).

### 2.8. Scenarios

We further simulated two scenarios based on the fitted model. Scenario 1 assumed that the interventions on 13 September and 28 September did not occur and that dengue fever spread at the same rate in the second and third periods as in the first period. Scenario 2 assumed no intervention on 28 September and the rate of dengue fever transmission in the third period was the same as that in the 13 to 28 September period.

### 2.9. Simulation Method

Coefficients *β_IS_*, *β_pcmc_*, *β_mcpc_*, *β_pcmo_*, *β_mopc_ β_pomo_* and *β_mopo_* were obtained by fitting the transmission models to the collected data with Berkeley Madonna 8.3.18 (developed by Robert Macey and George Oster of the University of California at Berkeley, CA, USA). The goodness of fit was assessed using the least root-mean-square error between the simulated and observed number of new cases per day. The coefficient of determination (R^2^), was assessed using SPSS 13.0 (IBM Corp, Armonk, NY, USA) to quantify the significance of the fit. In general, the fit was good when R^2^ is greater than 0.7. Make a map using Google Earth (Google Inc., Mountain View, CA, USA) software.

### 2.10. Normalization

In each model, we considered 7 transmission relative rates (*β_IS_* and so on); therefore, we fit each propagation coefficient separately for the case when the second and third cycles were zero, and then normalized the fitting results.

(3) $$\mathrm{Constituent}\;\mathrm{ratio}\;(y)=\frac{x_{other}}{x_{other}+x_{Chikan}}$$

(4) $$\mathrm{Normalized}=\frac{y-\min(y)}{\max(y)-\min(y)}$$

where *X_other_* is the number of cases in other districts (Mazhang, Xiashan and Potou district), *X_Chikan_* is the number of cases in Chikan district and *y* is the constituent ratio.

Based on the normalized results, we could compare the ability of different regions to spread the risk of disease and identify the districts with the highest risk of transmission. We defined that when the relative transmission rate was higher, the risk of disease transmission was higher.

## 3. Results

### 3.1. Reported Dengue Fever Cases in Zhanjiang City in 2018

In 2018, a total of 467 cases of DF were reported in Zhanjiang, including 447 indigenous cases and 20 imported cases, of which 15 and 5 were imported from domestic districts and overseas, respectively. In addition to the unrecorded serotypes, there were 222 cases with DENV 1, 7 cases with DENV 2 and 1 case with DENV 3. Overseas imports were from Cambodia (2 cases), Angola (1 case), Bangkok (1 case) and the Philippines (1 case). No deaths were reported. The annual incidence rate was 6.38 per 100,000 population. There were 234 clinically diagnosed and 233 laboratory-confirmed cases. The only DF outbreak appeared from late August to mid-October when cases spread from the construction sites to communities, and the outbreak was quickly contained after measures were taken to reduce the mosquito density.

### 3.2. Disease Distribution

**Figure 3:**
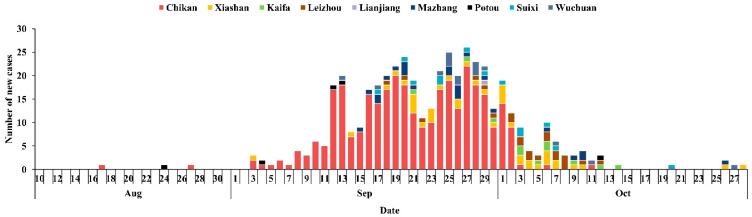
The outbreak of Dengue fever in Zhanjiang in 2018.

On 22 April, the first case of imported dengue fever in Zhanjiang occurred in Chikan District, and on 19 July, the first case of local dengue fever occurred in Xiashan District, and on 17 August, the first case of local dengue fever occurred in Chikan District. The number of cases was sporadically distributed in many places. The outbreak ended on 28 October (Figure 3).

After 17 August, the dengue fever outbreak spread to all areas of Zhanjiang, except Xuwen County. There were three main hotspots with 333 cases in Chikan District, 40 cases in Xiashan District and 23 cases in Ma Zhang District. The distribution of cases in other areas was 22 cases in Leizhou City, 15 cases in Wuchuan City, 13 cases in Suixi County, 11 cases in Kaihua District, 5 cases in Potou District and 1 case in Lianjiang City. Xiashan District and Chikan District had the longest presence of dengue fever with 56 days (Table 2).

**Figure 4:**
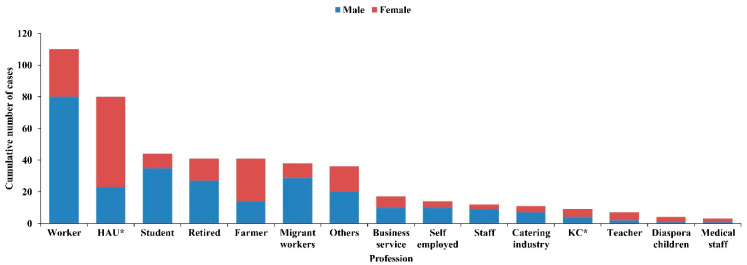
Profession distribution of dengue fever in Zhanjiang, Guangdong Province, China, in 2018 (HAU*: Housework an unemployed; KC*: Kindergarten children).

**Figure 5:**
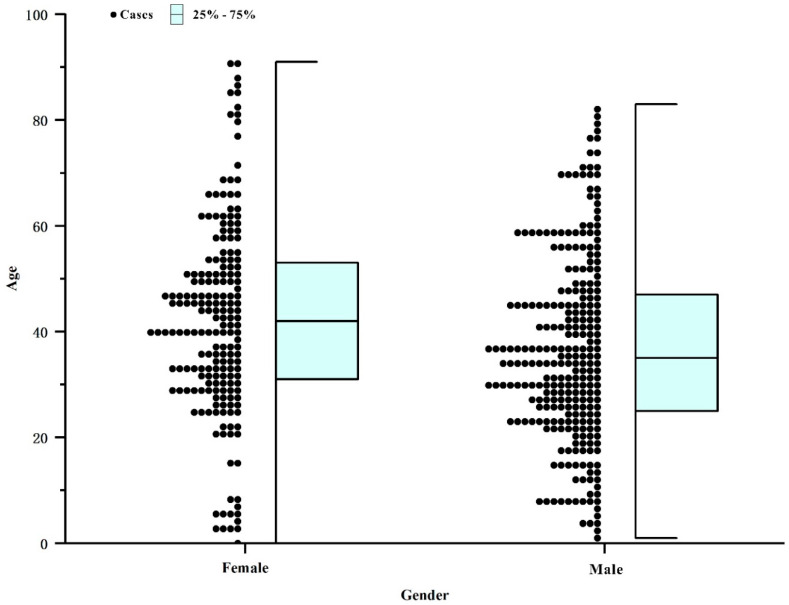
Gender distribution of dengue fever in Zhanjiang, Guangdong Province, China, in 2018. There were 272 males and 195 females, with a sex ratio of 1.39:1 and there was no significant difference in the sex ratio of cases (*p* > 0.05).

The 467 cases of dengue in 2018 involved multiple occupations. Workers (110 cases) accounted for the largest proportion (23.6%) of all cases, followed by household workers and the unemployed (80 cases, 17.1%), students (44 cases, 9.4%), the retired (41 cases, 8.8%) and farmers (41 cases, 8.8%) (Figure 4). The age distribution spanned from 6 months to 91 years old. Adults between the ages of 20 and 40 had the highest incidence (287 cases, 61%) (Figure 5).

### 3.3. Vector Surveillance

**Figure 6:**
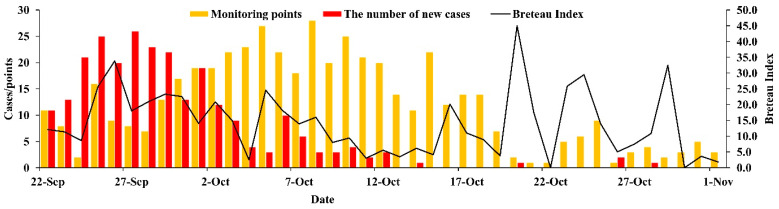
The number of new cases and the Breteau index in Zhanjiang in 2018.

**Figure 7:**
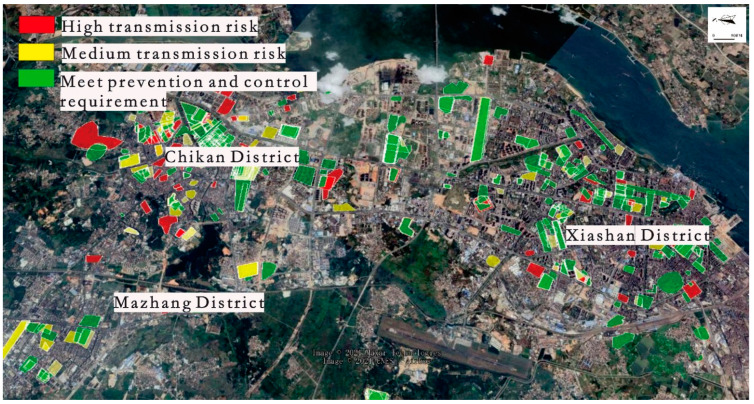
Distribution map of the Breteau index for dengue fever in Zhanjiang City in 2018.

Many districts in Zhanjiang City were monitored from 22 September to 1 November. The data showed that the overall level of the BI in Zhanjiang City was over 10 from 22 September to 8 October. The BI dropped quickly from 9 October. When the BI decreased below 5, the number of new cases was almost zero. The last case in this region appeared on 28 October (Figure 6). Daily monitor points of the Breteau index are also shown in Figure 6. According to the Breteau index, we divided the risk levels and present them in the form of a map in Figure 7.

### 3.4. Curve Fitting

The results of the curve fitting of the reported data showed that the simulated results agreed well with the reported data: R_Chikan_^2^ = 0.983 (*p* < 0.001), R_Kaifa_^2^ = 0.791 (*p* < 0.001), R_Mazhang_^2^ = 0.923 (*p* < 0.001) and R_Xiashan_^2^ = 0.956 (*p* < 0.001). The values of *β_IS_*, *β_pcmc_*, *β_mcpc_*, *β_pcmo_*, *β_mopc_ β_pomo_* and *β_mopo_* were generated via curve fitting and are summarized in Table 3.

### 3.5. Effectiveness of the Interventions

**Figure 8:**
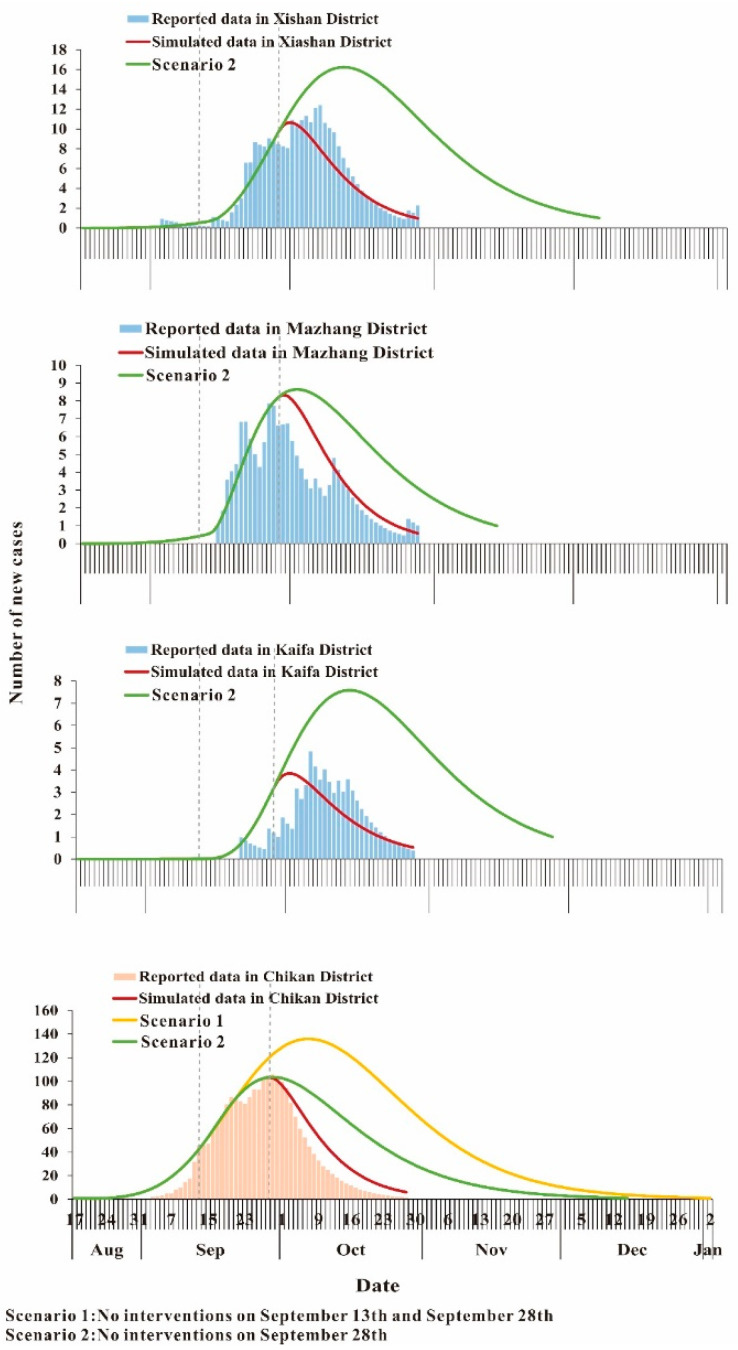
Curve fitting of dengue fever in Zhanjiang City, 2018.

In scenario 1 (Figure 8), the simulation results showed that in Chikan District, the number of existing cases continued to increase, peaking on 7 October and then slowly decreasing. In Kaifa District, there was no increase in cases after 13 September. In Ma Zhang District, cases slowly increased after 13 September and reached a peak on 7 October. In Xiashan District, the cases increased slowly after 13 September and declined slowly after 7 October.

In scenario 2, existing cases in Chikan District peaked on 29 September and then slowly declined. In Kaifa district, cases continued to increase after 28 September and peaked on 14 October. In Ma Zhang, cases began to decline after 2 October. In Xiashan, cases continued to rise after 28 September and declined after peaking on 12 October.

The simulation results showed that the number of new cases in each model was at least twice the actual number without any intervention, yielding an epidemic duration [27] of 113 days. In scenario 2, the number of new cases in each model was at least 1.4 times the observed data, yielding a DO of 95 (96) days (Table 4).

### 3.6. Comparison of Transmission Relative Rate

The normalization results showed that the *Nβ_IS_*, *Nβ_pcmc_*, *Nβ_mopc_* and *Nβ_pomo_* for the Chikan–Xiashan District transmission had the biggest values and the value of *Nβ_pcmo_* for the Chikan–Kaifa District transmission was the smallest (Table 5).

## 4. Discussion

Guangdong Province has been a high prevalence area for DF. We described the dengue fever epidemic in Zhanjiang City, Guangdong Province, in 2018 and developed an ODE model to fit it to the observed data in Zhanjiang County. The R^2^ results showed that our model fit the observed data well. The normalized results showed that dengue fever spread in the region. Government interventions brought the outbreak under control.

Young adults aged 20 to 40 years accounted for the majority of cases, which was probably because the outbreak started at construction sites where most of the workers were young adults. Occupationally, workers accounted for the largest proportion, followed by domestic workers and the unemployed, suggesting that more emphasis should be placed on educating them about dengue-related health issues. In addition, workers often work around the clock without protective measures or while wearing light clothing during the hot summer months, which may contribute to mosquito-borne diseases [27]. In subtropical cities, the minimum temperature and humidity during a month are positively correlated with the number of mosquitoes [28,29]. This explains why in our study, the only peak of dengue incidence occurred from late August to mid-October. The appropriate climate and high density of *Aedes* mosquitoes provide the conditions for epidemics to occur. Socioeconomic factors also played an important role in the epidemic [30,31]. The sanitary conditions at construction sites are poor, with domestic waste and construction materials scattered everywhere. Workers are accustomed to living together, which can cause rapid transmission in the event of a case. Residents of Zhanjiang are accustomed to storing water in tanks, which provides conditions for *Aedes* mosquitoes to breed and may contribute to the spread of the outbreak from the construction site to the community.

BI surveillance was conducted throughout the epidemic period. The high vector density provided a high receptivity, and therefore, a high probability for DF transmission. Since DF is a mosquito-borne disease, killing mosquitoes can lead to a decrease in the disease burden. When control measures, such as mosquito eradication, were taken, the BI was kept below the safety line and the epidemic was controlled, suggesting that the timely reduction of the *Aedes* mosquitoes density can contribute to effective prevention and control of the epidemic, which is a result that is also supported by our model.

In this study, the SEIAR model was used to fit the epidemic curves of the outbreak in Zhanjiang City. The results of the coefficient of determination showed a good fit of our model to the reported data, which indicated that the model could fit a similar situation, i.e., comparing the transmissibility of DF between different regions. In scenario 1, the number of dengue cases in Zhanjiang City would have more than doubled and the duration of the outbreak would have more than doubled if the government had not implemented any interventions and instead allowed the disease to spread naturally. When the government took control measures on 13 September (scenario 2), there was some relief. However, the number of cases and the duration were also significantly higher than the actual situation. As we can see, the timely intervention by the government effectively mitigated the duration of the epidemic and the number of infected people. This shows that timely and multiple preventive and control measures are necessary in the event of a dengue fever outbreak.

Among the four districts considered, Chikan district had the strongest internal transmission, which indicated that the transmission of dengue fever was strongest within Chikan district. This was because Chikan District is located in the central area of Zhanjiang City, with large population mobility and a huge population. Karl et al. said human activity can promote the spread of dengue [32,33]. A study by Poter et al. [34] in two textile mills showed that some workers were infected at the workplace and some at home, suggesting that controlling human activity is an important condition for controlling dengue. One potential problem could be the weather conditions in different areas of Zhanjiang City. Different local weather also has an impact on the local mosquito activity profile in different parts of the city [35].

Zhanjiang has a permanent resident population of 7.33 million with high mobility. The epidemic first occurred in Chikan District, then in Mazhang, Kaifa and Xiashan Districts. This chronological order suggested that there may have been inter-regional transmission of the dengue virus. The normalized results showed that dengue fever was transmitted within the districts. This suggested that the detection of a case in one district can serve as an early warning signal and that other districts should implement appropriate interventions. When the transmission of the dengue virus from Chikan to other districts was cut off, the number of cases in other districts was significantly reduced or even zero. When measures were taken to control the epidemic diseases in Chikan District in time, such as controlling the population movement between regions, case isolation, adult vector control and larvae control, the epidemic in other districts was alleviated or even stopped from breaking out. In addition, in the analysis of inter-regional transmission, the greatest human-to-mosquito transmissibility was from Chikan District to Mazhang District, where infected people moved from Chikan District to Mazhang District and infected local mosquitoes, suggesting that the risk of DF transmission between these two districts was greater than in other districts. Once an outbreak occurs, the movement of people between these two districts must be more strictly controlled. Furthermore, we found that the greatest transmissibility from mosquitoes to people was from people who were infected with Xiashan District mosquitoes to people in Chikan District, suggesting that stricter prevention and intervention measures must be implemented in these two areas in terms of controlling the density of adults and larvae.

### Limitations

Although we gained some insights from this research, we must recognize that the data used in this study were collected from the passive public health surveillance system. Reporting methods, under-reporting or misdiagnosis, and the availability of health facilities and laboratory diagnostics may have all affected the data quality. Moreover, some asymptomatic cases may not have visited the hospital, which was also not included in the surveillance system. Since there have been few dengue fever cases in Zhanjiang in the past few years, we used only one year of data to elucidate the pattern of transmission between districts. If data become available in the future, some more multidimensional and multi-year regional analyses can be done.

## 5. Conclusions

When a dengue outbreak occurs in one district, it spreads to other districts. Thus, a dengue outbreak in one district can serve as an early warning signal for other districts. The OED model can effectively model the spread of dengue between districts. Adopting the same level of control in all districts is not the best approach, and concentrating different control measures in different districts is the best way to control dengue outbreaks.

## Figures and Tables

**Table 1:** Parameter definitions and values.

Parameter	Description	Unit	Value	Range	Method
*a_c_/a_o_*	Daily birth rate of mosquitoes in Chikan district or other districts *^1^	day^−1^	0.0714	0.0200–0.2500	References
*c_c_/c_o_*	Seasonality parameter of the mosquitoes’ population in Chikan district or other districts	1	See Equation (2) *^2^	0–1	Curve fitting
*n_c_/n_o_*	Proportion of transovarial transmission in Chikan District or other districts	1	0.1	0.0140–0.1740	Reference
*b_c_/b_o_*	Daily death rate of mosquitoes in Chikan District or other districts	day^−1^	0.0714	0.0200–0.2500	Reference
*ω_m_/* *ω_p_*	Relative incubation rate of mosquito infection or human infection	day^−1^	0.1000/0.1667	0.0833–0.1250/	Reference
				0.1250–0.2500	
*q*	Proportion of asymptomatic infections	1	0.6875	0–1	Reference
*γ*	Relative removal rate of infectious individuals	day^−1^	0.1429	0.0714–0.3333	Reference
*γ’*	Relative removal rate of asymptomatic individuals	day^−1^	0.1429	0.0714–0.3333	Reference
τ	Simulation delay of the initial time in the whole season	day	279	≥0	Analysis of the reported data
T	Duration of the cycle	day	365	≥0	Analysis of the reported data
*β_IS_*	Relative transmission rate from imported mosquitoes to mosquitoes in Chikan District	1	See Table 2	≥0	Curve fitting
*β_mcpc_*	Relative transmission rate from mosquitoes in Chikan District to humans in Chikan District	1	See Table 2	≥0	Curve fitting
*β_mopc_*	Relative transmission rate from mosquitoes in other districts to humans in Chikan District	1	See Table 2	≥0	Curve fitting
*β_mopo_*	Relative transmission rate from mosquitoes in other districts to humans in other districts	1	See Table 2	≥0	Curve fitting
*β_pcmc_*	Relative transmission rate from humans in Chikan District to mosquitoes in Chikan District	1	See Table 2	≥0	Curve fitting
*β_pcmo_*	Relative transmission rate from humans in Chikan District to mosquitoes in other districts	1	See Table 2	≥0	Curve fitting
*β_pomo_*	Relative transmission rate from humans in other districts to humans in other districts	1	See Table 2	≥0	Curve fitting

*^1^ Subscript *c* indicates Chikan District, subscript *o* indicates other districts (Kaifa, Xiasha and Mazhang District). *^2^ The parameter *c* was simulated with the trigonometric function using the parameters *τ* and *T*.

**Table 2:** Regional distribution of dengue fever outbreak in Zhanjiang.

Variable	Male	Female	Total	Duration (Days)
District				
Chikan	200	133	333	56
Xiashan	22	18	40	56
Leizhou	10	12	22	25
Mzhang	13	10	23	42
Wuchuan	6	9	15	45
Suixi	5	8	13	34
Kaifa	8	3	11	23
Potou	4	1	5	50
Lianjiang	0	1	1	1
Total	268	195	463	-

**Table 3:** Parameter values.

Parameter	Chikan–Kaifa	Chikan–Mazhang	Chikan–Xiashan
*β_IS_*	1.73 × 10^−9^	2.23 × 10^−9^	1.53 × 10^−9^
*β_pcmc_*	3.19 × 10^−8^	4.13 × 10^−8^	3.10 × 10^−8^
*β_mcpc_*	1.86 × 10^−5^	1.49 × 10^−5^	1.94 × 10^−5^
*β_mopc_*	7.88 × 10^−10^	1.18 × 10^−9^	7.31 × 10^−10^
*β_pomo_*	5.16 × 10^−16^	6.72 × 10^−16^	5.30 × 10^−16^
*β_pcmo_*	5.21 × 10^−8^	2.55 × 10^−8^	2.57 × 10^−8^
*β_mopo_*	5.67 × 10^−7^	1.66 × 10^−6^	1.70 × 10^−6^

**Table 4:** Scenario fitting results.

District	Fit		Scenario 1			Scenario 2		
	Number of New Cases	Duration	Number of New Cases	Proportion *	Duration	Number of New Cases	Proportion *	Duration
Chikan	410	56	974	137.56%	114	581	41.71%	95
Kaifa	12	23	-	-	-	43	258.33%	37
Chikan	406	56	932	129.56%	114	579	42.61%	95
Xiashan	34	55	-	-	-	97	185.29%	54
Chikan	406	56	917	125.86%	113	586	44.33%	96
Mazhang	27	41	-	-	-	43	59.26%	33

*: The increase ratio of the number of new cases in the two scenarios to the fitted data.

**Table 5:** Normalized results.

Relative Transmission	Chikan–Kaifa District	Chikan–Xiashan District	Chikan–Mazhang District
*N* *β_IS_*	0.441441	0.463294	0.389146
*N* *β_pcmc_*	0.449167	0.464421	0.39408
*N* *β_mcpc_*	1	1	1
*N* *β_mopc_*	0.441441	0.463294	0.389146
*N* *β_pomo_*	0.441441	0.463294	0.389146
*N* *β_pcmo_*	0.064007	0.211337	0.389146
*N* *β_pcmo_*	0	0	0

## Data Availability

Data supporting the conclusions of this article are included within the article.
